# Amplifying undetectable NMR signals to study host–guest interactions and exchange†
†Electronic supplementary information (ESI) available: Experimental details are given in the ESI and include: sample preparations, NMR experiments, and DFT computational methods. See DOI: 10.1039/c6sc04083g



**DOI:** 10.1039/c6sc04083g

**Published:** 2016-10-05

**Authors:** Liat Avram, Mark A. Iron, Amnon Bar-Shir

**Affiliations:** a Department of Chemical Research Support , Weizmann Institute of Science , 7610001 Rehovot , Israel; b Department of Organic Chemistry , Weizmann Institute of Science , 7610001 Rehovot , Israel . Email: amnon.barshir@weizmann.ac.il

## Abstract

The characteristics of host–guest systems, such as molecular recognition, complexation, encapsulation, guest composition, and dynamic exchange, are manifested by changes in the chemical shifts (Δ*ω*) in the NMR spectrum. However, in cases where NMR signals cannot be detected, due to low concentrations, poor solubility, or relatively fast exchange, an alternative is needed. Here, we show that by using the magnetization transfer (MT) method, the undetectable NMR signals of host–guest assemblies can be amplified by two orders of magnitude. It is shown that the binding kinetics characteristics of a fluorinated guest and cucurbit[*n*]uril (CB[*n*]) hosts in aqueous solutions determine the NMR signal amplification of host–guest assemblies. In addition, by using the MT technique within the ^19^F-NMR framework, one can detect μM concentrations of the complex and study the effect of different solutes on the resulting host–guest system. The results expand the “NMR toolbox” available to explore a wider range of dynamic host–guest systems in which NMR signals cannot be detected.

## Abstract 2

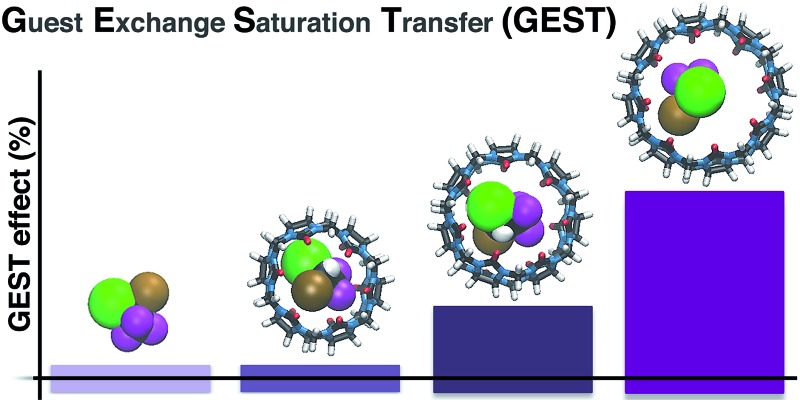
Undetectable NMR signals of host–guest assemblies can be amplified by two orders of magnitude using the proposed GEST methodology.

## Introduction

Host–guest interactions are at the core of an endless number of supramolecular systems comprising complexes of molecules that, through non-covalent interactions, are held together in a three-dimensional assembly. Among the developed and studied host–guest assemblies, water-soluble systems have garnered much interest due to their potential applications.^1–5^ While the hydrophilicity of the outer sphere of the host makes it water soluble, the hydrophobicity of its cavity allows for the inclusion of guests. This capacity has been exploited, for example, for generating confined hydrophobic spaces,^6,7^ synthetic cyclization,^8,9^ drug delivery,^10^ molecular switches,^11^ molecular imaging,^12^ anion receptors^13^ and theranostic systems.^14^

The desired property of the supramolecular assembly is determined, and can be tuned, by the binding interactions between the molecular guest and the binding cavity of its three-dimensional host. The host–guest assemblies can be characterized using isothermal titration calorimetry (ITC), X-ray crystallography, optical measurements, mass spectrometry or nuclear magnetic resonance (NMR) spectroscopy. Of these analytical tools, NMR has been extensively exploited, and is often the technique of choice, for studying and characterizing supramolecular assemblies in solution.^15,16^ The chemical shifts (Δ*ω*) in the NMR spectrum depend on the chemical environment and reflect molecular structures, moiety interactions, assemblies, dynamicity, temperature, pH and other properties of the environment. Therefore, resolving the NMR spectra is vital to the study of supramolecular systems, and the inability to identify certain chemical shifts may dramatically impact the interpretation of the results. Specifically, when studying host–guest interactions, by resolving characteristic Δ*ω* one can determine (i) guest entrapment, (ii) location/composition of the guest within the host, (iii) binding constants, and (iv) molecular recognition. However, the low sensitivity and low signal-to-noise ratio (SNR) of conventional NMR methodologies are major limitations when detecting very low-concentration targets (*e.g.*, due to poor solubility or for a specific application). Moreover, weak host–guest interactions and/or dynamic exchange processes may cause NMR line-broadening that further severely reduces the SNR. Therefore, solutions are required to expand the capabilities of resolving Δ*ω*.

Magnetization transfer (MT, see ESI for a detailed explanation†) is an NMR technique in which a pool of NMR-observable nuclei (^1^H, ^19^F, ^13^C, ^31^P, *etc.*) is magnetically labeled (at a specific NMR frequency) using saturation or an inverse pulse, followed by a “label” transfer to a second pool of nuclei.^17–20^ In the specific case of a dynamic exchange process between two pools of nuclei, the radiofrequency pulse is applied at the chemical shift (Δ*ω*) of the low-concentration pool. The magnetization of this pool is nullified (“labeled”) and transferred to the pool of nuclei with higher concentration through an exchange process. When the exchange rate (*k*_ex_) is sufficiently fast (but still fulfills the condition of Δ*ω* > *k*_ex_), an MT effect can be detected through the reduction of the signal of the high-concentration pool. This facilitates the detection of a pool of spins at very low concentration, below the typically reasonable concentrations used for NMR studies, with the sensitivity of the high concentration pool. This has been demonstrated in a wide range of applications from molecular MRI^21–23^ to hyperpolarized ^129^Xe in host–guest systems.^24–26^ Here we propose using this technique to study dynamic host–guest interactions with a conventional NMR setup.

In this study, cucurbit[*n*]uril (CB[*n*])^27,28^ host molecules and a ^19^F-molecular guest were used to demonstrate the NMR signal amplification of a few host–guest systems at μM concentrations. The well-defined structure of CB[*n*], their cavity rigidity, their unique host–guest recognition capabilities, water solubility and biocompatibility make them ideal for many host–guest studies.^29–32^ By using the ^19^F-MT methodology in a conventional NMR setup, we demonstrate 100-fold signal amplification, and we can monitor NMR-undetectable signals and specific interactions between a fluorinated guest (2-bromo-2-chloro-1,1,1-trifluoroethane = halothane) and CB[8]. The signal amplification of >600-fold-diluted CB[8] offers a new method for the study of host–guest interactions where the NMR signals of the complex cannot be detected.

## Results and discussion

**Scheme 1:**
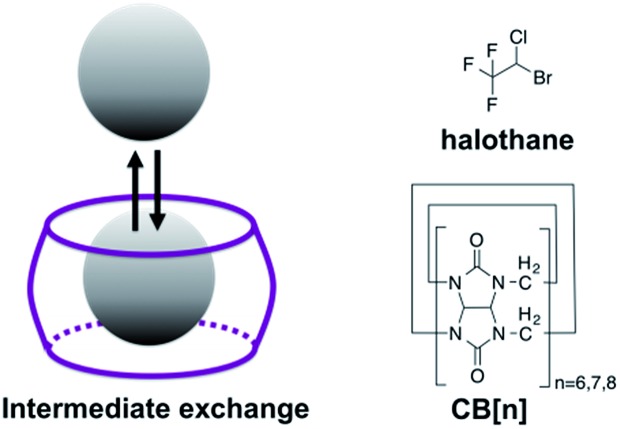
Left: Schematic illustration of the dynamic exchange between the encapsulated and free guest. Right: Structures of the host (CB[*n*]) and guest (halothane) used in this study.

**Fig. 1:**
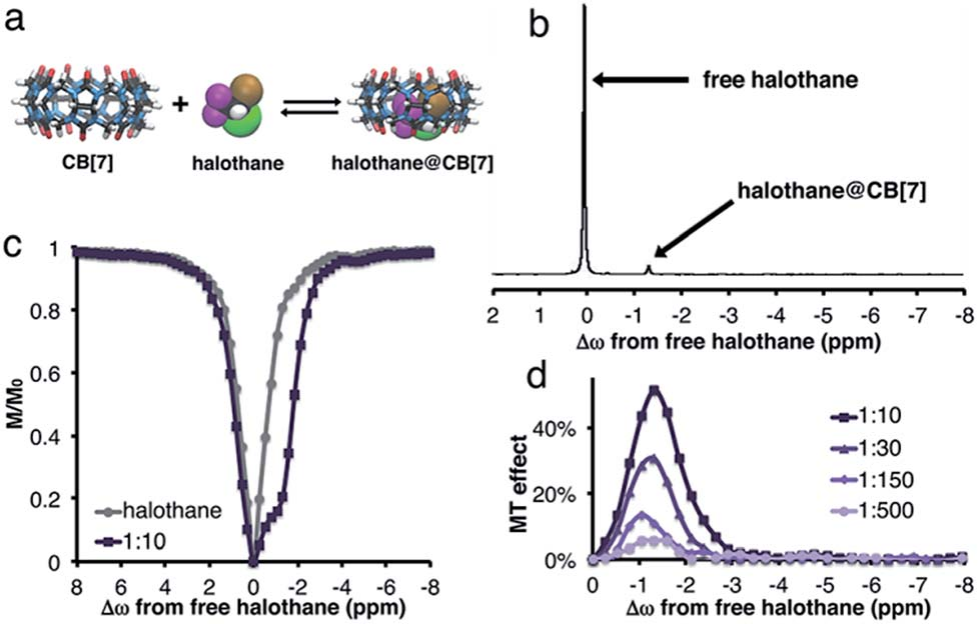
CB[7]:halothane host–guest system in D_2_O. (a) DFT optimized structures of CB[7], halothane, and the halothane@CB[7] complex. (b) ^19^F-NMR spectrum of 5 mM halothane and 0.5 mM CB[7] in D_2_O. (c) Plot of the relative ^19^F-NMR signal of halothane as a function of the frequency of the applied saturation pulse (*i.e.*, *z*-spectrum). (d) MT effect for CB[7]:halothane solutions with various molar ratios.

Scheme 1 depicts the dynamic exchange process between a free and a CB[*n*]-encapsulated fluorinated guest. For such an exchange to occur, the guest should be both soluble in the aqueous solution as well as stable in the hydrophobic cavity of the host. For such purposes, halothane – a fluorinated anesthetic that is soluble both in aqueous solutions and in lipids^33,34^ – was selected as the potential guest. The use of a ^19^F-guest permits the application of ^19^F-NMR, which has a greater sensitivity of the chemical shift to the environment (compared to ^1^H-NMR) as reflected in the larger chemical shifts of fluorinated guests upon encapsulation.^35,36^ Furthermore, MT within the ^19^F-NMR framework will emphasize the exchange effects and reduce other effects (such as NOE) that would be obtained with the more popular ^1^H-NMR.^37^ Fig. 1a shows the density function theory (DFT) optimized structures of CB[7], halothane, and the halothane@CB[7] complex. Clearly, it is feasible for CB[7] to accommodate halothane. As shown in Fig. 1b, in addition to the peak of free halothane, a second peak is observed in the ^19^F-NMR spectrum, 1.3 ppm upfield from free halothane (Δ*ω* = –1.3 ppm), which can be assigned to the entrapped halothane in CB[7]. Note that the ^19^F-NMR spectra were acquired in the presence of an internal reference, which was used for the calibration of the chemical shifts (see ESI†). In order to determine the dynamic exchange properties between the free and CB[7]-entrapped guest, MT experiments were performed and are summarized in Fig. 1c and d (for information regarding the MT experiments and the data analysis, see the ESI†).

**Fig. 2:**
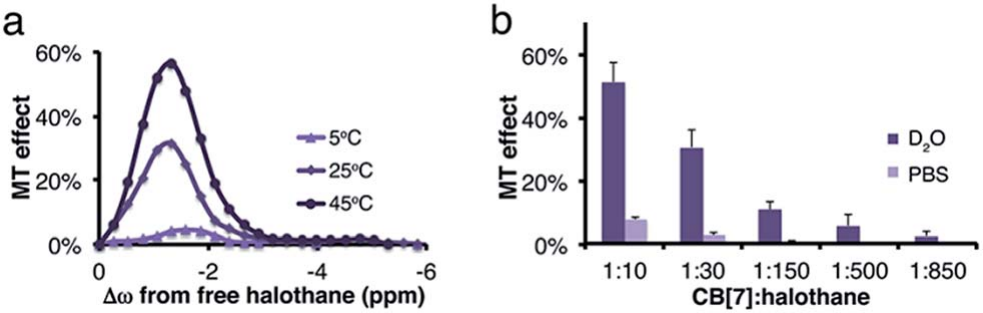
(a) Temperature dependence of the MT effect for a 1 : 30 molar ratio of CB[7] : halothane in D_2_O. (b) Comparison of MT effect (calculated at Δ*ω* = –1.3 ppm from free halothane) of CB[7]:halothane solutions at different molar ratios in D_2_O and PBS.

While no MT effect was observed in the solution that contained only halothane, a huge effect was measured in the presence of CB[7], with a maximum at the chemical shift of bound halothane. By reducing the concentration of CB[7], resulting in an increased host : guest molar ratio between the halothane (guest) and CB[7] (host), a reduction in the MT effect was observed, a phenomenon that is expected in two-pool exchange systems (Fig. 1d).^38^ This ability to transfer magnetization from a diluted host–guest complex (*i.e.*, 10 μM of CB[7]–halothane complex), and still obtain information about the chemical shift (Δ*ω* = –1.3 ppm, Fig. 1d) of the encapsulated guest, allows for the detection of low-concentration complexes with conventional NMR instrumentation. In order to demonstrate the effect of the dynamic exchange between free and CB[7]-encapsulated halothane, the temperature dependence of the MT effect was examined (Fig. 2a). As expected, as the temperature was elevated from 25 °C to 45 °C, the MT effect increased, which is consistent with a faster exchange between the free and encapsulated halothane. Likewise, reducing the temperature to 5 °C almost eliminated the MT effect. Some differences in the temperature-dependent ^19^F-NMR spectra can be observed (Fig. S1, ESI†), but the effect is much more pronounced in the MT experiments (Fig. 2a).

The effects of dissolved salts on host–guest interactions, including CB[*n*]s, have been discussed elsewhere.^39,40^ We found that the MT values obtained from CB[7]–halothane solutions are also affected by the salt content in the solution (Fig. 2b). For instance, at a host : guest molar ratio of 1 : 10, the effect is seven times higher in D_2_O than in a phosphate buffer saline (PBS) solution. Moreover, while an MT effect is still observed at a 1 : 850 host : guest ratio (2.3 ± 1.4%) in D_2_O, it was unobservable at a molar ratio >1 : 30 in PBS solutions. These findings again show the uniqueness and strength of the proposed approach to determine host–guest binding kinetics. While conventional ^19^F-NMR fails to identify differences in the exchange kinetics (Fig. S2, ESI†), the MT method detects the significant impact of salt content on the exchange processes.

**Fig. 3:**
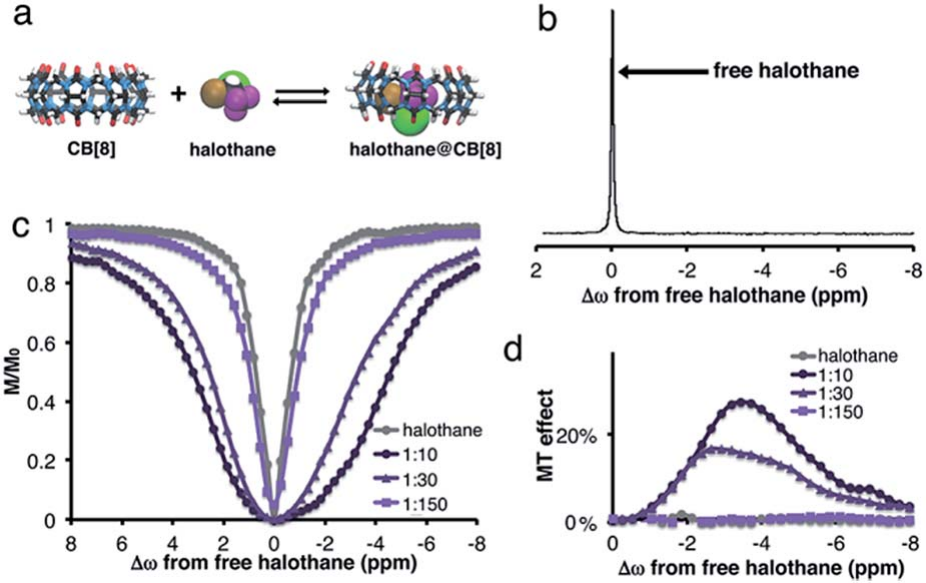
CB[8]:halothane host–guest system in D_2_O. (a) DFT optimized structures of CB[8], halothane and halothane@CB[8]. (b) ^19^F-NMR spectrum of 2 mM halothane and 0.2 mM CB[8] in D_2_O. (c) Relative ^19^F-NMR signal of halothane as a function of the frequency of the applied saturation pulse (*i.e.*, *z*-spectrum). (d) MT effect for CB[8]:halothane solutions with various molar ratios.

The effect of host size on the complexation and dynamic exchange processes was evaluated using other CB[*n*] hosts. The DFT optimized structures of CB[8], halothane, and the halothane@CB[8] complex are shown in Fig. 3a. In this case, no additional peak of halothane@CB[8] can be detected in the ^19^F-NMR spectrum (Fig. 3b). Therefore, it might be concluded, erroneously, that halothane does not bind to CB[8].

Interestingly and surprisingly, there is clear broadening of the MT plot in experiments performed on CB[8]:halothane solutions, with the maximum MT effect obtained at Δ*ω* = –3.4 ppm (Fig. 3d). This upfield chemical shift of the bound halothane may reflect the encapsulation of the fluorinated guest within the hydrophobic cavity of CB[8]. Such a determination cannot be achieved from conventional ^19^F-NMR, but is readily apparent in MT experiments. Note that by using an internal ^19^F-reference (see ESI†), we could determine that there is no change in Δ*ω* of free halothane upon addition of CB[8]. The absence of a bound halothane peak in the ^19^F-NMR spectrum (Fig. 3b) is probably due to faster exchange between free and bound halothane. Such an intermediate exchange limits the efficiency of the saturation pulse,^41^ and thus reduces the MT effect (compare Fig. 1d and 3d). Both observations (for CB[7]–halothane in Fig. 1 and for CB[8]–halothane in Fig. 3) are supported by DFT calculations (Fig. S3, see ESI for further details†), where higher barriers for halothane decomplexation are found for CB[7]–halothane (Δ*E* ^‡^ = 22.1 kcal mol^–1^) than CB[8]–halothane (Δ*E* ^‡^ = 11.5 kcal mol^–1^). The lower Δ*E* ^‡^ calculated for the CB[8]–halothane system supports the hypothesis of faster exchange in the CB[8] system.

**Fig. 4:**
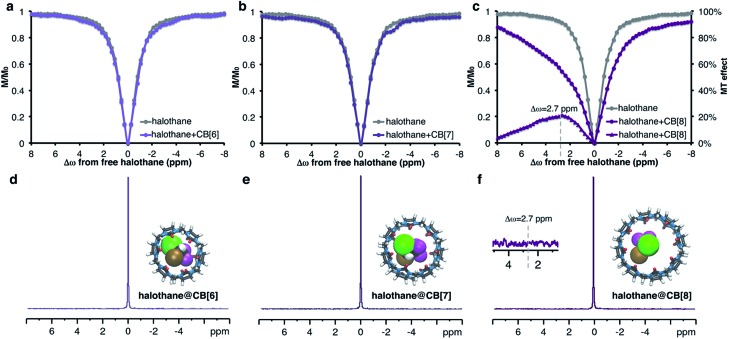
^19^F-MT *vs.* ^19^F NMR of the CB[*n*]:halothane system in PBS with a molar ratio of 1 : 600. (a–c) ^19^F *z*-spectra of halothane (gray circles) and halothane + CB[*n*] (purple circles). (d–f) ^19^F NMR spectra of the halothane + CB[*n*] samples. In (c), the MT effect (purple triangles, right *y*-axis) is also shown. The insets in panels (d–f) show the DFT optimized structures of the halothane@CB[*n*] complex. Each point in the ^19^F-MT spectra (panels a–c) represents a ^19^F NMR spectrum acquired with NS = 8. The ^19^F NMR spectra in (d–f) were acquired with NS = 128. The inset in panel f shows the magnification of the ^19^F NMR spectrum region where the signal of halothane@CB[8] is expected.

It was observed that PBS slows the exchange rates with CB[7] (*vide supra* Fig. 2b). Thus, this approach was attempted with other members of the CB[*n*] family (*n* = 6–8, Fig. 4 and S4, ESI†). Using CB[*n*] : halothane molar ratios of 1 : 50, no MT effect was observed for CB[6] (Fig. S4, ESI†). This is probably because its cavity size is too small to accommodate halothane; DFT calculations support this premise (see ESI†). For CB[7] at this molar ratio, only a minuscule effect is detected (See Fig. S4, ESI†). Due to the poor solubility of CB[8] in PBS, we prepared low concentration (10 μM) solutions of CB[*n*], resulting in a 1 : 600 (CB[*n*] : halothane) molar ratio. Surprisingly, despite the very low concentration of the host, an enormous MT effect (approx. 20%) was detected (Fig. 4c). This observation can only be explained by a dynamic exchange process between CB[8]-entrapped and free halothane. Such an exchange process is generally manifested by line-broadening and a reduced NMR signal, which is apparent in the ^1^H NMR spectra of CB[8] and CB[8]:halothane (Fig. S5, ESI†).

All of the complexes studied so far show upfield MT effects. Nevertheless, halothane@CB[8] in PBS surprisingly has a maximum MT effect at Δ*ω* = +2.7 ppm downfield from free halothane (Fig. 4c purple triangles). This downfield shift may indicate that the orientation of the guest molecule within the host – specifically the relative positions of the CF_3_ groups – is different in D_2_O and PBS. Since the ^19^F NMR peak of the CB[8]-bound halothane could not be detected in either D_2_O or PBS, this conclusion can only be reached using ^19^F-MT experiments. It is important to note that the 20% change in the ^19^F NMR signal was observed using a minimal number of scans (NS = 8 per ^19^F NMR spectrum). In contrast, even with 128 scans, no evidence of halothane–CB[8] interactions could be observed in the ^19^F-NMR spectrum (Fig. 4f, inset).

It should be mentioned here that although the hyperpolarized-^129^Xe MT approach has been used to study host–guest interactions, including CB[*n*] with ultra-high sensitivity,^24–26^ by using a fluorinated guest, one can now use ^19^F NMR to study a wider range of supramolecular systems. Moreover, these experiments can be performed using standard NMR spectrometers without the additional dedicated hardware required for hyperpolarized experiments.

**Fig. 5:**
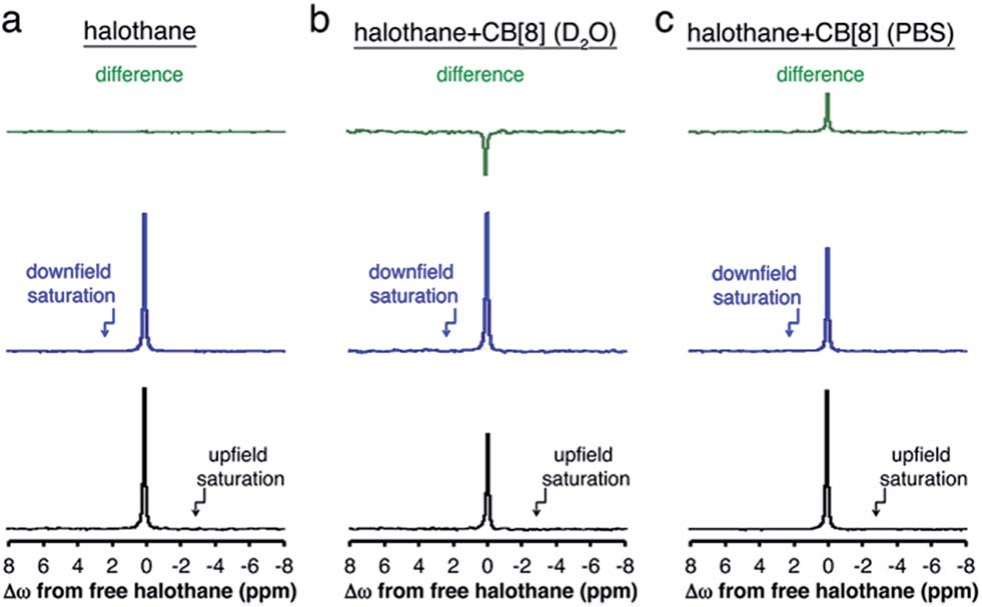
^19^F NMR spectra of (a) halothane in D_2_O, (b) halothane + CB[8] in D_2_O, and (c) halothane + CB[8] in PBS with B_1_ (saturation pulse) applied either downfield (blue spectra) or upfield (black spectra) of free halothane. The differences between the spectra (residual signal) are shown above in green.

To summarize the opposite chemical shift offset for the host–guest system of halothane–CB[8] obtained in either D_2_O or PBS, the ^19^F-NMR spectra acquired with presaturation pulses are shown in Fig. 5. Also shown are the residual peaks (green spectra) obtained after subtraction of the ^19^F-NMR spectrum acquired with a presaturation pulse applied upfield (black spectra) and downfield (blue spectra) of the frequency offset of free halothane. While no residual peak was found for the aqueous solution containing only halothane (no MT effect, Fig. 5a, *M* ^+Δ*ω*^ = *M* ^–Δ*ω*^), opposite residual peaks are obtained for halothane–CB[8] in D_2_O (Fig. 5b, *M* ^+Δ*ω*^ > *M* ^–Δ*ω*^) and PBS (Fig. 5c, *M* ^+Δ*ω*^ < *M* ^–Δ*ω*^).

## Conclusions

In conclusion, we have demonstrated the performance of the MT approach in the ^19^F NMR framework to study dynamic host–guest interactions. By capitalizing on the dynamic exchange process between the free and bound ^19^F-guest, the magnetization from a few μM (>600-fold-diluted solution) of CB[8]-hosted halothane could be transferred to the high-concentration free halothane (a few mM), and this allowed the detection of otherwise NMR-undetectable ^19^F-moieties. This ability to detect low-concentration complexes through the NMR signal of the high-concentration free guest, using a minimum number of NMR scans and a conventional NMR setup, allows for the detection of host–guest complexes and interactions that could not be characterized by routine NMR methodologies. This capability enabled the observation of a unique phenomenon in which the salt content of the solution changed Δ*ω* of the complexed guest from upfield to downfield relative to the free guest using a typical NMR setup without varying the concentrations of either the host or the guest. The combination of ^19^F-guests, together with the MT-based approaches for NMR, can be extended to study a wider range of supramolecular systems. Analogously to the chemical exchange saturation transfer (CEST) used for molecular MR imaging, the proposed approach may be termed GEST – guest exchange saturation transfer. The GEST approach will play a pivotal role in understanding the dynamics of host–guest molecular systems.
