# Supplementary material for: Amplifying undetectable NMR signals to study host–guest interactions and exchange
Source: Chem Sci. 2016 Oct 5;7(12):6905–9. doi: 10.1039/c6sc04083g (PMC5450594; doi:10.1039/c6sc04083g)
Supplement: Supplementary file 1 [file SC-007-C6SC04083G-s001.pdf]

Electronic Supplementary Material (ESI) for Chemical Science.
This journal is © The Royal Society of Chemistry 2016

Amplifying undetectable NMR signals to study host-guest interactions and exchange

Liat Avram[1], Mark A. Iron[1] and Amnon Bar-Shir[2]*

[1]Department of Chemical Research Support and [2]Department of Organic Chemistry,

Weizmann Institute of Science, 7610001 Rehovot, Israel

Supporting Information

Experimental Section

Chemicals: The cucurbit[n]urils, (CB[n], n = 6-8), were purchased from Strem Chemicals UK, Ltd. Halothane (2-bromo-2-chloro-1,1,1-trifluoroethane) was purchased from Sigma Aldrich. Phosphate Buffer Saline (PBS) was purchased from ThermoFisher Scientific.

Sample preparation: CB[n]s were dissolved in either D_2O or PBS@D_2O solution to the desired final concentration (as mentioned in the main text). To a 4 mL aqueous solution of the studied CB[n], ^{19}F-halothane was added to obtain halothane:CB[n] samples. To determine the final concentration of the halothane in the sampled solution (placed in a 5 mm NMR tube), a capillary containing a known concentration and a volume of KF in D_2O was inserted into the NMR tube. The integration values of both fluorine-containing compounds (^{19}F-halothane:unknown and F^- from KF: known concentration) were used to determine the absolute concentration of the ^{19}F-halothane in the sample. The molar ratio between the guest (halothane) and the host (CB[n]) was then calculated for each sample.

NMR experiments: All NMR experiments were performed on a 9.4 T NMR spectrometer (Bruker, Germany), with the sample temperature stabilized at 298 K. *^{1}H-NMR spectra* (400 MHz) were acquired for all samples prior to the ^{19}F-NMR experiments. Number of scans (NS): 128. *^{19}F-NMR* (376.7 MHz) spectra were acquired with 128 scans. *T_1 and T_2 of halothane*: The longitudinal (T_1) and transverse (T_2) relaxation times of halothane were calculated using inversion recovery (IR) and Car-Purcell-Meiboom-Gill (CPMG) experiments, and were found to be 6 sec and 1.5 sec, respectively.

MT experiments (^{19}F-z-spectra): ^{19}F-magnetization transfer (MT) data was acquired as follows: a pre-saturation pulse with a length of either 15 sec or 3 sec was applied prior to the 90° rf pulse. The saturation pulse strength B_1 was set to 150 Hz. The frequency of the presaturation pulse was swept from $\Delta\omega$ = +8.2 ppm to $\Delta\omega$ = -8.2 ppm offset (in 100 Hz = 0.26 ppm steps) relative to the resonance frequency of free halothane (which was set to 0 ppm for convenience). In addition, a ^{19}F-NMR spectrum where the rf presaturation pulse was applied at $\Delta\omega$ = +65 ppm was acquired as a reference spectrum. For each frequency offset, the data was acquired with eight scans, using a repetition time of 15 sec, resulting in an experimental time of *approx.* 2 min per ^{19}F-NMR spectrum and a total experiment time (of all 64 saturation frequencies) of 2 h 13 min. Then

the z-spectrum was plotted for each experiment. The normalized signal ($M^{\Delta\omega i}/M^0$) at each frequency offset was plotted as a function of the frequency offset of the presaturation pulse to obtain a characteristic z-spectrum. The MT effect was calculated from the signal intensities of each spectrum ($M^{\Delta\omega i}$): $MT_{effect} = 100 \times (M^{-\Delta\omega} - M^{+\Delta\omega})/M^{+65ppm}$ was computed for different Δω offsets.

Density Function Theory (DFT) calculations: All calculations were done using GAUSSIAN09 REVISIONS D.01 and E.01.[1] Geometries were optimized with the Perdew-Burke-Ernzerhof (PBE) functional,[2, 3] with an added empirical dispersion correction,[4-7] specifically the third version of Grimme's dispersion[4, 8] with Becke-Johnson dampening;[8-10] this combination is denoted as PBE_{D3BJ}. For geometry optimizations, the def2-SVP basis set[11, 12] was used, which includes a relativistic effective core potential (RECP) on bromine. When using a GGA functional, such as PBE, density-fitting basis sets were used in order to accelerate the calculations,[13, 14] specifically the def2SV fitting basis set that complements def2-SVP.[12] The reaction profiles in **Figure S2a-c** were obtained as rigid scans: starting from the optimized geometry, the center of mass of the halothane was translated to the center of mass of the halothane (only a small step from the optimal geometry) and then translated along the axis perpendicular to the CB[n] cage. A special script was written to prepare the geometries. Energies were then calculated using Truhlar's Minnesota-06 hybrid functional – specifically, M06-2X[15] – which has been shown to be very reliable for the barrier heights; the third version of Grimme's dispersion[4, 8] with a zero-order dampening was added. The def2-TZVP basis set[11, 12] was used. To avoid gird errors, as recommended by Wheeler and Houk when using functionals of the Minnesota-06 family, the "ultrafine" (99,590) pruned integration grid was used.[16] Bulk solvent effects were approximated by single-point energy calculations, using a polarizable continuum model (PCM);[17-20] specifically, the integral equation formalism model (IEF-PCM).[17, 18, 21, 22] Truhlar's empirically parameterized version, Solvation Model Density (SMD), was used.[23]

Supplementary Figures

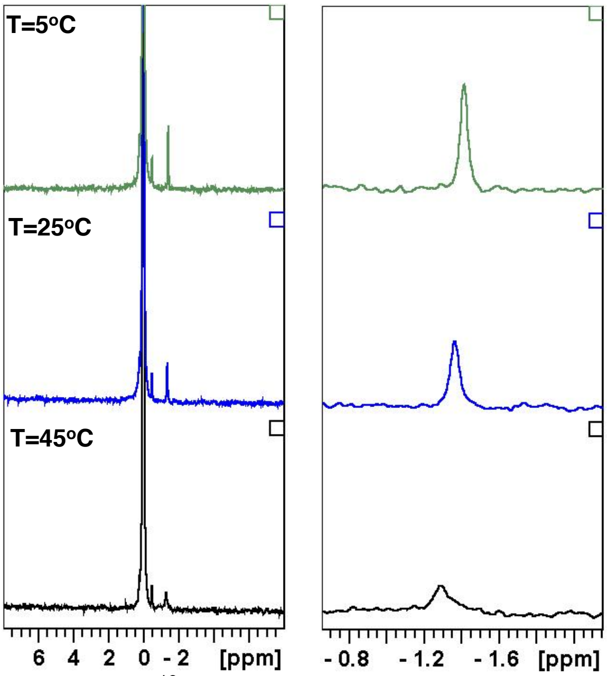

Figure S1. ^{19}F-NMR spectra (376.7 MHz) of CB[7]:halothane 1:30 solution (in D_2O) as a function of temperature. Spectra were acquired with 128 scans.

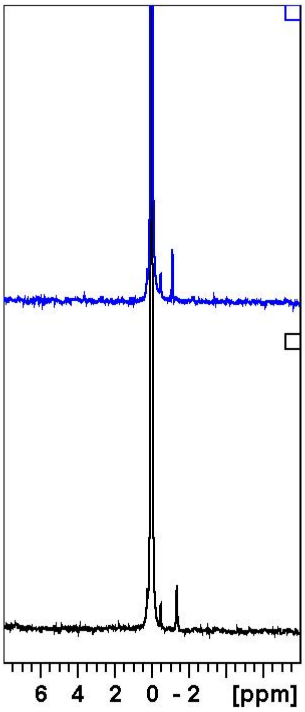

Figure S2. ^{19}F-NMR spectra (376.7 MHz) of CB[7]:halothane 1:30 solutions in D_2O (black spectrum) and in PBS (blue spectrum). Spectra were acquired at 298K with 128 scans.

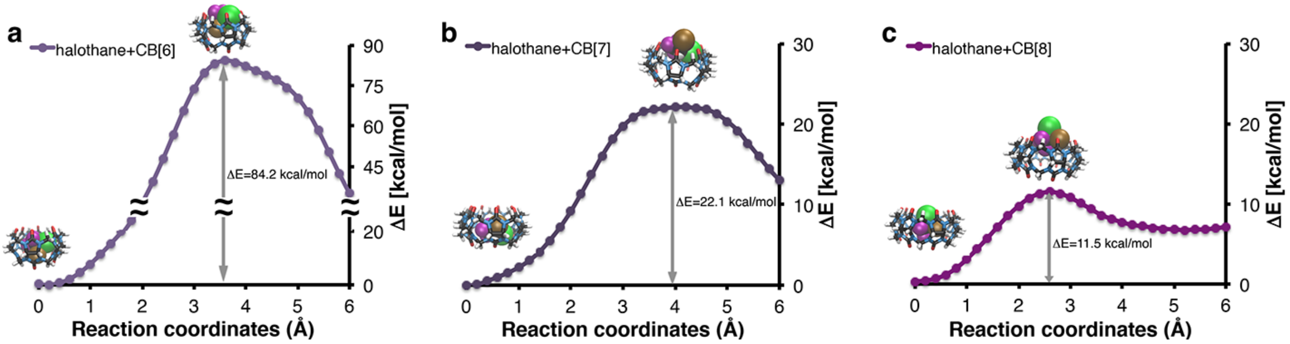

Figure S3. DFT reaction profiles for removal of halothane from (a) CB[6], (b) CB[7] (b) and (c) CB[8]. Shown are the molecular structures of the halothane@CB[n] complexes (minimum energy, i.e., ΔE = 0 kcal/mol) and of the corresponding transition states (maximum energy, i.e., ΔE = 84.2 kcal/mol for halothane@CB[6], ΔE = 22.1 kcal/mol for halothane@CB[7], and ΔE = 11.5 kcal/mol for halothane@CB[8]).

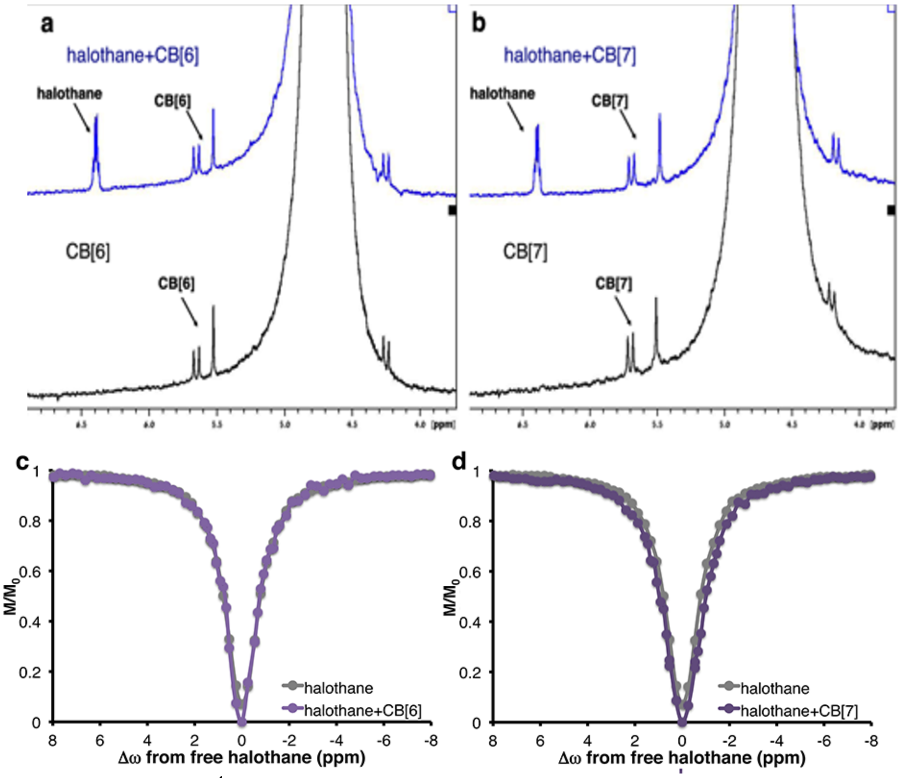

Figure S4. (a,b) ^{1}H-NMR spectra (400 MHz, 298K, 128 scans) of PBS aqueous solutions of 50 μM CB[n] (black spectra) and CB[n]:halothane (blue spectra 1:50 molar ratio) (a: n = 6; b: n = 7). (c,d) Plots of the relative ^{19}F-NMR signal of halothane (grey) and halothane+CB[n] (purple) in PBS as a function of the frequency of the applied saturation pulse. (c: n = 6; d: n = 7)

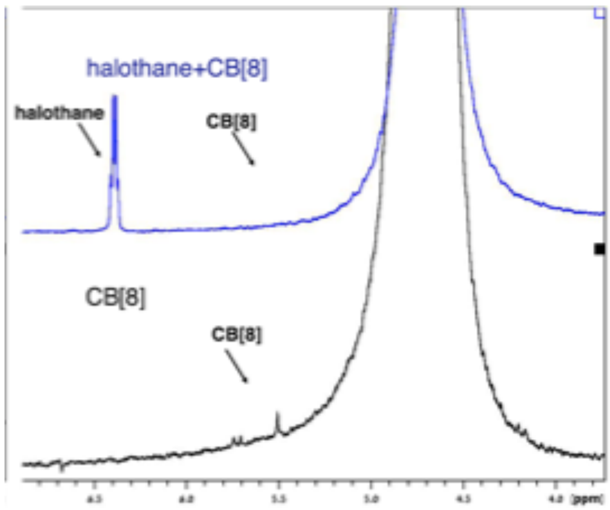

Figure S5. ^{1}H-NMR spectra (400 MHz) of PBS aqueous solutions of (i) 10 μM CB[8] (black spectra) and CB[8]:halothane (blue spectra 1:600 molar ratio). ^{1}H-NMR spectra were acquired at 298K with 128 scans.

^{19}F-MT NMR experiments and data analysis

By using magnetization transfer (MT) experiments, one can transfer magnetization between two pools of exchanging nuclear spins. This system can be used for molecular systems where a nuclear spin is reversibly transferred between two non-equivalent sites. The exchangeable molecule (in our case CB[n]-bound halothane) is "magnetically tagged" by specific radiofrequency pulses (**Scheme S1a**, Saturation). The saturated ("labeled") halothane exchanges with free halothane and affects the net magnetization of the surrounding free halothane, thus influencing its ^{19}F-NMR signal intensity (ΔSI, **Scheme S1b**, Detection). If the exchange process between bound and free halothane is sufficiently fast and the applied saturation pulse is sufficiently long, this approach enables the detection of very low concentrations of bound halothane through the change in the signal intensity of the high concentration free halothane.

The experimental data is plotted as shown in **Scheme S1c,d**. The presaturation RF pulse is applied at different frequencies upfield and downfield to the frequency of free halothane (set at 0 ppm for convenience). The signal intensity is then plotted as function of the frequency offset (**Scheme S1c**). To reduce effects of direct saturation of the free halothane peak and to get the net MT effect, the signal intensities of each spectrum, $MT_{effect} = 100 \times (M^{-\Delta\omega} - M^{+\Delta\omega})/M^{0}$, are computed at different $\Delta\omega$ offsets.

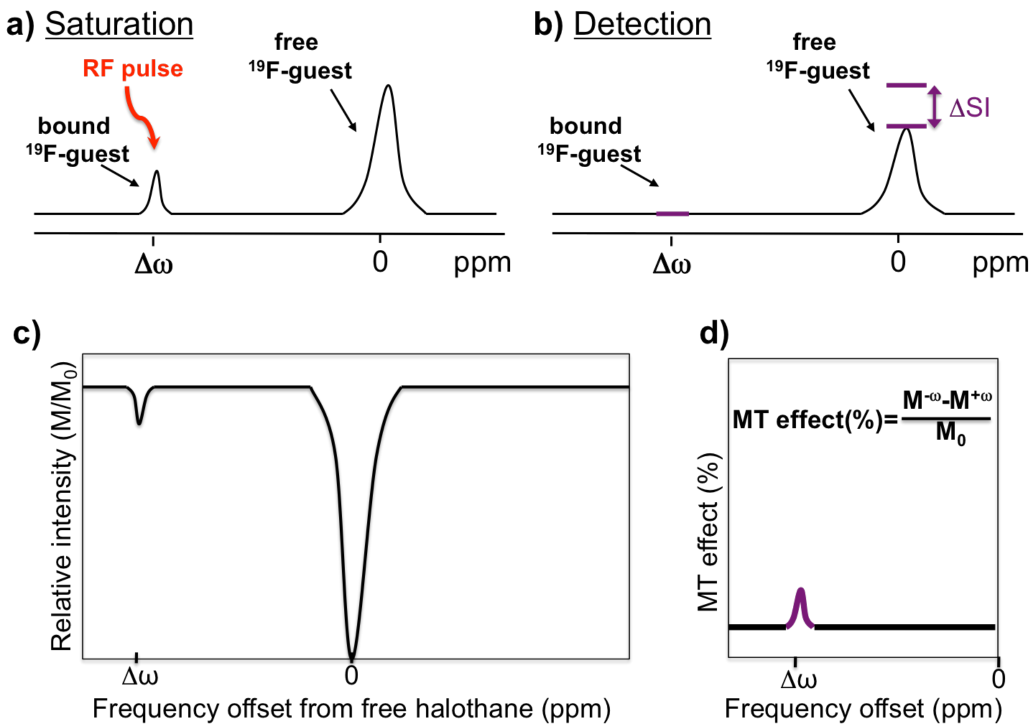

Scheme S1: A frequency selective saturation pulse (Saturation) is applied (a) and the reduction in the signal of free guest, ΔSI, is detected (b, Detection). (c) Signal intensity as function of the frequency offset of the applied RF pulse. (d) The calculated MT effect.

References

1. Gaussian 09, Revision D.01, M. J. Frisch, G. W. Trucks, H. B. Schlegel, G. E. Scuseria, M. A. Robb, J. R. Cheeseman, G. Scalmani, V. Barone, B. Mennucci, G. A. Petersson, H. Nakatsuji, M. Caricato, X. Li, H. P. Hratchian, A. F. Izmaylov, J. Bloino, G. Zheng, J. L. Sonnenberg, M. Hada, M. Ehara, K. Toyota, R. Fukuda, J. Hasegawa, M. Ishida, T. Nakajima, Y. Honda, O. Kitao, H. Nakai, T. Vreven, J. A. Montgomery, Jr., J. E. Peralta, F. Ogliaro, M. Bearpark, J. J. Heyd, E. Brothers, K. N. Kudin, V. N. Staroverov, T. Keith, R. Kobayashi, J. Normand, K. Raghavachari, A. Rendell, J. C. Burant, S. S. Iyengar, J. Tomasi, M. Cossi, N. Rega, J. M. Millam, M. Klene, J. E. Knox, J. B. Cross, V. Bakken, C. Adamo, J. Jaramillo, R. Gomperts, R. E. Stratmann, O. Yazyev, A. J. Austin, R. Cammi, C. Pomelli, J. W. Ochterski, R. L. Martin, K. Morokuma, V. G. Zakrzewski, G. A. Voth, P. Salvador, J. J. Dannenberg, S. Dapprich, A. D. Daniels, O. Farkas, J. B. Foresman, J. V. Ortiz, J. Cioslowski, and D. J. Fox, Gaussian, Inc., Wallingford CT, 2013.
2. J. P. Perdew, K. Burke and M. Ernzerhof, *Phys. Rev. Lett.*, 1996, **77**, 3865-3868.
3. J. P. Perdew, K. Burke and M. Ernzerhof, *Phys. Rev. Lett.*, 1997, **78**, 1396.
4. S. Grimme, J. Antony, S. Ehrlich and H. Kreig, *J. Chem. Phys.*, 2010, **132**, 154104.
5. T. Schwabe and S. Grimme, *Acc. Chem. Res.*, 2008, **41**, 569-579.
6. T. Schwabe and S. Grimme, *Phys. Chem. Chem. Phys.*, 2007, **9**, 3397-3406.
7. S. Grimme, *J. Comput. Chem.*, 2006, **27**, 1787-1799.
8. S. Grimme, S. Ehrlich and L. Goerigk, *J. Comput. Chem.*, 2011, **32**, 1456-1465.
9. E. R. Johnson and A. D. Becke, *J. Chem. Phys.*, 2006, **124**, 174104.
10. E. R. Johnson and A. D. Becke, *J. Chem. Phys.*, 2005, **123**, 024101.
11. A. Schäfer, H. Horn and R. Ahlrichs, *J. Chem. Phys.*, 1992, **97**, 2571-2577.
12. F. Weigend and R. Ahlrichs, *Phys. Chem. Chem. Phys.*, 2005, **7**, 3297-3305.
13. B. I. Dunlap, *J. Chem. Phys.*, 1983, **78**, 3140-3142.
14. B. I. Dunlap, *J. Mol. Struct. (THEOCHEM)*, 2000, **529**, 37-40.
15. Y. Zhao and D. G. Truhlar, *Theor. Chem. Acc.*, 2008, **120**, 215-241.
16. S. E. Wheeler and K. N. Houk, *J. Chem. Theory Comput.*, 2010, **6**, 395-404.
17. B. Mennucci and J. Tomasi, *J. Chem. Phys.*, 1997, **106**, 5151-5158.

18. E. Cancès, B. Mennucci and J. Tomasi, *J. Chem. Phys.*, 1997, **107**, 3032-3041.
19. M. Cossi, V. Barone, B. Mennucci and J. Tomasi, *Chem. Phys. Lett.*, 1998, **286**, 253-260.
20. M. Cossi, G. Scalmani, N. Rega and V. Barone, *J. Chem. Phys.*, 2002, **117**, 43-54.
21. B. Mennucci, E. Cancès and J. Tomasi, *J. Phys. Chem. B*, 1997, **101**, 10506-10517.
22. J. Tomasi, B. Mennucci and E. Cancès, *J. Mol. Struct. (THEOCHEM)*, 1999, **464**, 211-226.
23. A. V. Marenich, C. J. Cramer and D. G. Truhlar, *J. Phys. Chem. B*, 2009, **113**, 6378-6396.
